# Biometric Physiological Responses from Dairy Cows Measured by Visible Remote Sensing Are Good Predictors of Milk Productivity and Quality through Artificial Intelligence

**DOI:** 10.3390/s21206844

**Published:** 2021-10-14

**Authors:** Sigfredo Fuentes, Claudia Gonzalez Viejo, Eden Tongson, Nir Lipovetzky, Frank R. Dunshea

**Affiliations:** 1 Digital Agriculture Food and Wine Group, School of Agriculture and Food, Faculty of Veterinary and Agricultural Sciences, University of Melbourne, Parkville, VIC 3010, Australia; cgonzalez2@unimelb.edu.au (C.G.V.); eden.tongson@unimelb.edu.au (E.T.); fdunshea@unimelb.edu.au (F.R.D.); 2 School of Computing and Information Systems, Melbourne School of Engineering, University of Melbourne, Parkville, VIC 3010, Australia; nir.lipovetzky@unimelb.edu.au; 3 Faculty of Biological Sciences, The University of Leeds, Leeds LS2 9JT, UK

**Keywords:** heart rate, respiration rate, abrupt movements, robotic dairy farm, artificial neural networks

## Abstract

New and emerging technologies, especially those based on non-invasive video and thermal infrared cameras, can be readily tested on robotic milking facilities. In this research, implemented non-invasive computer vision methods to estimate cow’s heart rate, respiration rate, and abrupt movements captured using RGB cameras and machine learning modelling to predict eye temperature, milk production and quality are presented. RGB and infrared thermal videos (IRTV) were acquired from cows using a robotic milking facility. Results from 102 different cows with replicates (*n* = 150) showed that an artificial neural network (ANN) model using only inputs from RGB cameras presented high accuracy (R = 0.96) in predicting eye temperature (°C), using IRTV as ground truth, daily milk productivity (kg-milk-day^−1^), cow milk productivity (kg-milk-cow^−1^), milk fat (%) and milk protein (%) with no signs of overfitting. The ANN model developed was deployed using an independent 132 cow samples obtained on different days, which also rendered high accuracy and was similar to the model development (R = 0.93). This model can be easily applied using affordable RGB camera systems to obtain all the proposed targets, including eye temperature, which can also be used to model animal welfare and biotic/abiotic stress. Furthermore, these models can be readily deployed in conventional dairy farms.

## 1. Introduction

The global dairy industry growth has been steadily increasing in recent years, shown by the worldwide milk volume, reaching 513.23 million metric tons in 2020. In addition, global milk production is forecasted to increase by 22% in 2027 [1] and is projected to increase 2.3 fold (1168 million tons) by 2030 [2]. Australia and New Zealand are two counties currently dominating the international dairy trade with 12% and 32% market representation, respectively [3]. In Australia, dairy production represents one of the most important rural industries, with a market value of AUD 3.2 billion in 2018–2019 [4]. Consequently, the dairy industry has focused on improving milk yield and quality by researching and implementing technologies that allow the identification and reduction of factors that hinder the performance of dairy cattle. Several challenges related to climate change and logistics are expected to impact the dairy industry worldwide, such as (i) consumer concerns about animal welfare, (ii) warming environment and effect on animal welfare and productivity, (iii) increased resource costs and (iv) high-cost labor-related issues [5]. Different strategies have been devised to help increase the efficiency of dairy resource management and, consequently, the efficiency of production and quality traits of milk produced [5].

Robotic-milking systems (RMS) have been developed to improve the efficiency and productivity of milk in dairy farms and have become a valued asset in many farms around the world [6]. The benefits of this system have promoted the implementation of RMS in close to 35,000 farms worldwide by 2019 [7]. The main advantage of this system is the possibility that cows have to voluntarily approach the automatic milking machines at any time of the day using feeding as a reward [8]. It has been observed that this voluntary approach generates an increase in milking frequency [9,10]. Rodenburg [11] observed that cattle milking frequency in RMS was on average three or more visits per day compared to an average of 2.5 visits using the conventional milking systems [12]. Consequently, milk yield improved compared to farms that use a more traditional milking system [13]. Furthermore, RMS has also shown differences in the feeding behavior of cows when compared to conventional milking systems. For example, greater cow feeding activity occurred during the daytime and early evening hours (9 a.m. to 3 p.m. and 5 p.m. to 1 a.m.) compared to late night and early mornings (1 a.m. to 7 a.m.), which is also normally observed in other milking systems [14].

In addition to the improvements in milking systems, great effort has also been devoted to improving cow monitoring on dairy farms, predominantly non-invasive or less intrusive, as health and wellbeing significantly impact cows and their productivity performance [15]. Stress has been recognized as one of the factors affecting the animal production of meat and milk. In this matter, dairy cows are constantly exposed to several stressors, such as social interactions (animal–animal and human–animal interactions), environmental stressors (heat stress) and novelty and physical restraint, among others [16]. Several responses to these stressors have been researched and used as indicators of stress levels in cattle [15,16]. Although behavioral, adrenocortical and physiological responses have been defined as stress indicators in farm animals, behavioral and adrenocortical responses have been suggested to be limited by their high degree of stressor specificity and their assessment methods [17,18]. In the case of physiological responses, these are mainly driven by two axes: the hypothalamic–pituitary–adrenal (HPA) and the sympathetic–adrenal–medullary (SAM) [19]. The SAM axis generates a more rapid response to the stress stimuli, involving metabolic, physiologic and immunologic changes in the animal [20]. For instance, heart rate, breathing rate and skin temperature increase due to stressful situations [20,21]. Although physiological responses to stress have become common in animal monitoring, their assessment still includes some invasive methods that can produce animal stress and pain, affecting final animal wellbeing results through biases [21,22].

As previously mentioned, physiological parameters have been commonly measured through contact techniques (highly and moderately invasive). For instance, heart rate (HR) in cattle has been widely measured by using stethoscopes [23], electrocardiograms (ECG) and some commercial monitors such as Polar^®^ Heart Rate Monitors (Polar Electro Oy, Kempele, Finland) [24,25,26]. Moreover, the body temperature of cattle has been assessed by techniques that can measure rectal, vaginal, ruminal and skin temperature, among others. Rectal temperature has been the most common technique to measure body core temperature, performed using a thermometer [27,28,29] or thermometric probe [30]. Although these techniques have been validated and greatly implemented in the investigation of the physiological changes generated by a stressful situation or environment in cattle, the requirement for human–animal contact for insertion and data handling make these techniques less accurate and impractical for large scale assessment due to biases associated to the actual stress given to animals by the method and potentially poor capacity for repeatability. Hence, the development of new technologies has taken place in animal welfare research to produce reliable, affordable and accurate non-invasive methods to assess these physiological parameters in animals [31,32].

Researchers are currently implementing remote sensing and computer vision to monitor and detect behavioral and physiological changes that could reflect health and wellbeing issues in animals in a less invasive way. This has been done by implementing algorithms that can analyze imagery (visible and thermal) and identify animal behavior, posture and corporal condition, among others [33,34,35]. Furthermore, infrared thermal imagery (IRTI) and visible or red, blue and green (RGB) images have been used to assist the assessment of emotion changes in humans [36,37,38,39] and animals with great success, as presented in this study. In cattle, the measurement of eye temperature from IRTIs has been used to relate to stress levels in several situations [40]. For instance, Gómez, Bieler, Hankele, Zähner, Savary and Hillmann [18] used eye temperature extracted from IRTIs as non-invasive physiological indicators of acute stress in cows when they are exposed to stressful situations. Other methods, such as biometric techniques, have been used to identify physiological changes and emotion detection in humans. For instance, Eulerian Video Magnification has been tested in humans to measure heart rate (HR) and heart rate variability (HRV) [41]. Other algorithms with increased accuracy have also been developed and used to detect changes in physiological parameters and emotions in human consumers when exposed to several products [38]. The implementation of these techniques have been published by Torrico, et al. [42] and Gonzalez Viejo et al. [36,37], using customized algorithms to measure skin temperature and HR as responses to several food stimuli in human consumers through non-invasive video analysis and IRTI.

All techniques mentioned above for humans are based on computer vision algorithms to automatically track specific features from RGB videos and extract information from regions of interest (ROI). Since RGB videos and IRTIs can be obtained in parallel using either two cameras or an integrated camera, as done in this research, these images can be reregistered to extract data from RGBs and IRTIs from animals at the same time. Therefore, this study aimed to implement computer vision algorithms developed to extract information from videos and IRTIs on dairy cows and to construct a machine learning model based on regression fitting to predict eye temperature (°C), using IRTV as ground truth, daily milk productivity (kg-milk-day^−1^), cow milk productivity (kg-milk-cow^−1^), milk fat (%) and milk protein (%) with no signs of overfitting.

The developed models based on data from conventional RGB cameras could be used in conventional and robotic dairy farms. The latter will allow the implementation of artificial intelligence techniques to target specific volume/quality of milk and automatically identify and manipulate stressors or stressful environments within the farm, such as heat stress using misters or shelters [43].

## 2. Materials and Methods

### 2.1. Study Site and Animals Description

**Figure 1:**
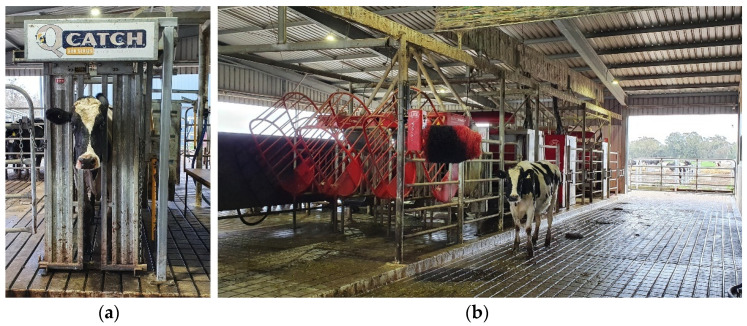
Robotic dairy facility used for this study showing (**a**) the crush where the cows were restrained for visible and infrared thermal video (IRTV) recording and (**b**) robotic milking area to the left of crush where the cows were redirected after data collection.

This study was approved by the Animal Ethics Committee of The University of Melbourne (Ethics ID: 2021-21466-18833-5). The experiment was conducted on four dates in a southern hemisphere winter (14–15 July and 4–5 August 2021) from 9 am to 4 pm in the robotic dairy facilities located at The University of Melbourne Dookie College, Victoria, Australia (36°38′ S, 145°71′ E). These facilities consist of three Lely Astronaut robotic milking units (Lely Holding S.à.r.l., Maassluis, The Netherlands) with laser-guided teat detection, which can milk up to 180 cows a day. The system works on semi-voluntary milking where cows are motivated by individually formulated supplementary feed and an automatic Lely Luna brush for cows’ comfort. A total of 102 different and non-stressed Holstein-Friesian cows with one to five replicates for the analyses were used for this study (*n* = 282). The replication per cow varies because the cows assessed per day were based on semi-voluntary milking, as previously explained, and were not forced to participate in the study. When cows approached the facilities for milking, some of these were directed to the crush before and others after milking (Figure 1a) to be recorded, as explained in Section 2.2. If the cows showed signs of high stress, they were released and excluded from that measurement to comply with ethics regulations. Once the recording was over, they were redirected to the milking area either for milking or to obtain some individually formulated supplementary feed (Figure 1b). This approach of having some cows milked before or after the recording was made to avoid bias due to the milking factor.

### 2.2. Video Recording and Physiological Measurements—Biometrics

**Figure 2:**
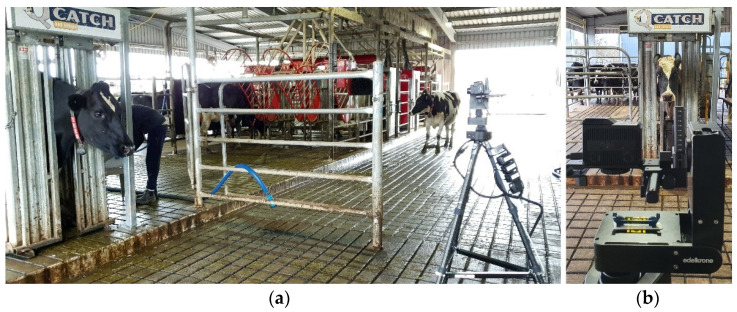
Visible and infrared thermal (IRTV) recording showing (**a**) the position of the camera mounted on a tripod facing the cows in the crush and (**b**) frontal view of a cow being recorded.

A visible red, green and blue (RGB) and infrared thermal camera FLIR DUO PRO (Teledyne FLIR LLC, Wilsonville, OR, USA) was mounted on a Benro Tortoise 34C carbon fiber tripod (Guangdong Benro Image Technology Industrial Co., Ltd., Zhongshan City, Guangdong Province, China) loaded with a PAN PRO and a HeadPLUS motorized pan and tilt device (Edelkrone USA, Inc., Winter Park, FL, USA) 2.5 m away from the crush and facing the cows (Figure 2). The Pan PRO was used to adjust and lock the camera’s position, while the HeadPLUS was used to tilt the camera 10° to completely record the crush and center the cow’s head. 4K Videos were recorded for 1 min per cow. Single RGB and infrared thermal frames had a resolution of 2160 × 3840 and 514 × 652 pixels, respectively.

The regions of interest (ROI) from the RGB videos were selected and tracked using the point tracker based on the Kanade–Lucas–Tomasi (KLT) algorithm in the video labeller application in Matlab^®^ Computer Vision Toolbox 10.0 (Mathworks Inc., Natick, MA, USA). The selected ROIs were the eye section to assess heart rate (HR) and the nose to determine respiration rate (RR). The labels generated from the video labeller were used to crop the videos using a customized code written in Matlab^®^ R2021a (Mathworks, Inc., Natick, MA, USA) developed by the Digital Agriculture Food and Wine (DAFW) group from The University of Melbourne (UoM), Australia.

**Figure 3:**
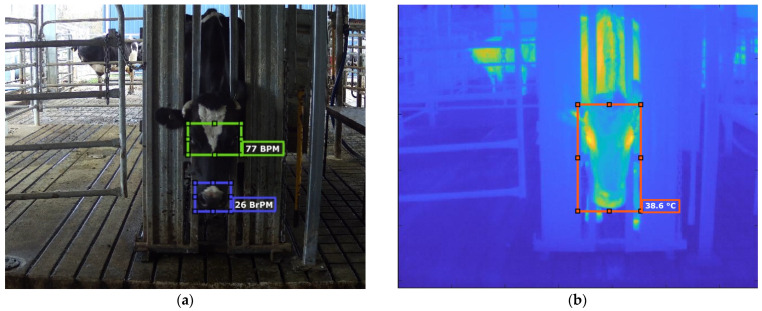
Example of a frame obtained and analysis from (**a**) a visible RGB video showing the regions of interest used to obtain heart rate (eye section, yellow rectangle) and respiration rate (nose, blue rectangle) showing the mean values after analysis displayed for a specific cow and (**b**) an infrared thermal video (IRTV) showing the region of interest used to analyze eye temperature and the mean value displayed for a specific cow.

The HR in beats per minute (BPM) was estimated from the eye section of the cropped videos. The HR algorithm developed in Matlab^®^ R2018a and updated/adapted in version R2021a by the DAFW-UoM group is based on luminosity changes in the green color channel from ROIs obtained, which uses the photoplethysmography (PPG) principle based on the peak analysis of the signal obtained from luminosity over time, which computes the amplitude and frequency of this signal [38,44] (Figure 3a). Furthermore, the RR in breaths per minute (BrPM) was computed using the copped videos from the nose section. The DAFW-UoM group developed the algorithm in Matlab^®^ R2020a for RR analysis in sheep [44] and was adapted for dairy cows in Matlab^®^ R2021a. This algorithm works similar to the HR PPG principle but uses a channel (green to red) from the CIELab color scale (Figure 3a).

The FLIR DUO PRO camera records the infrared thermal videos (IRTV) as sequence (.seq) files, which were analyzed using the SENSE Batch software (SENSE Software, Warsaw, Mazowsze, Poland) to extract the radiometric data per frame in batch and saved as comma-separated values (csv) files. These data were used to create the thermal videos as Motion Picture Experts Group Layer 4 (.MP4) using a customized Matlab^®^ 2021a code developed by the DAFW-UoM group. These videos were further used to detect and track the cows face using the point tracker based on the KLT algorithm in the video labeler application in Matlab^®^ Computer Vision Toolbox 10.0. The labelled IRTV and radiometric data were used to extract the maximum face temperature (eyes) using a computer vision algorithm developed in Matlab^®^ R2020a by the DAFW-UoM group (Figure 3b).

The abrupt movement analysis was based on the whole head tracking (Figure 3b, ROI rectangle) to automatically obtain its centroid as x-y coordinates to track head movements. From the entire video (1 min cow^−1^), four quartiles were analyzed to record patterns of movements within the length of the video. The cow did not move much from its original position if the x-y centroid coordinates were similar through the four quartiles. If x or y varied significantly, the cow’s head moved up and down (*y*-axis) or sideways (*x*-axis). The labels obtained from the ROI used in the IRTV analysis were analyzed using an algorithm developed by the DAFW-UoM group in Matlab^®^ 2021a; this algorithm works as previously described and extracts metrics and statistics automatically.

### 2.3. Weather Information

The weather information was obtained from the Dookie College Weather Station (Adcon Telemetry GmbH, Klosterneuburg, Austria) recorded every 15 min. The extracted data consisted of (i) temperature (°C; T), (ii) relative humidity (%; RH), (iii) wind speed (km h^−1^) and (iv) wind direction (°). The dew point temperature (T_dp_) was calculated using Equation (S1) in the Supplementary Material. This, along with T and surface pressure, was used to calculate the wet-bulb temperature (T_wet_) using a Matlab^®^ R2021a algorithm based on the bisection search method. The calculated T_dp_ and T_wet_, RH and T were used to calculate the temperature–humidity index (THI) using nine different equations (Supplementary Material Equations (S2)–(S10)) as previously described by Fuentes et al. [43].

### 2.4. Milk Production and Composition Data

The cows wear an identification transponder neck collar (Lely Holding S.à.r.l., Maassluis, The Netherlands) to record their activity and associated milk productivity and composition. Therefore, for this study, the data extracted consisted of (i) milk production per day (kg), (ii) milk production per milking (kg), (iii) milk fat (%) and (iv) milk protein (%).

### 2.5. Statistical Analysis and Machine Learning Modelling

Means and standard error (SE) from all parameters obtained from the physiological measurements, weather and milk data were computed, grouping the cows by age (2–7 years old). Furthermore, a multivariate data analysis based on principal component analysis (PCA) was developed using Matlab^®^ 2021a to assess relationships between the different physiological responses, milk data and wind speed.

**Figure 4:**
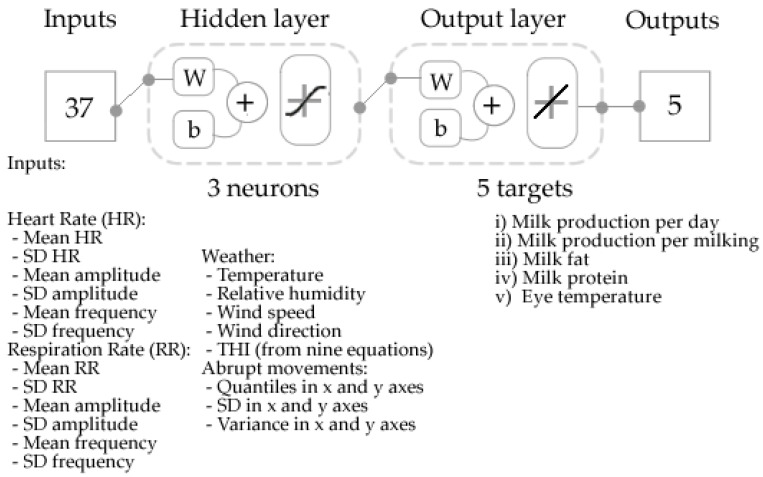
Diagram showing the two-layer feedforward model with tan-sigmoid function (hidden layer) and linear transfer function (output layer). Abbreviations: W: weights; b: bias.

An artificial neural network (ANN) model was developed using a code written by the DAFW-UoM group (Matlab^®^ R2021a), which can automatically test 17 different training algorithms to find the best model based on accuracy (correlation coefficient: R) and performance (means squared error: MSE) with no signs of under/overfitting. The Bayesian regularization was selected as the best training algorithm using the data from the physiological parameters obtained from the RGB videos (HR and RR), the weather information and abrupt movements in x and y axes (Figure 4). The targets for the model consisted of milk production (per day and per milking), milk composition (fat and protein) and eye temperature based on the IRTV. Apart from the performance, a condition to avoid under or overfitting of a model is to keep the number of inputs at <70% of the number of samples, which this model meet with 37 inputs <70% of samples (105). From *n* = 282 cow recordings, 150 were randomly selected to develop the model, while the remaining 132 were used for deployment. To develop the model, samples were divided randomly as 70% (*n* = 105) for training and 30% (*n* = 45) for testing. A neuron trimming test (3, 5, 7 and 10 neurons) was conducted to find the best model with no signs of under/overfitting.

The deployment was conducted by evaluating the model with data from the remaining 132 cow samples and conducting linear regression to assess the accuracy of the model’s outputs with respect to the observed data.

## 3. Results

### 3.1. Physiological, Weather and Milk Parameters

**Figure 5:**
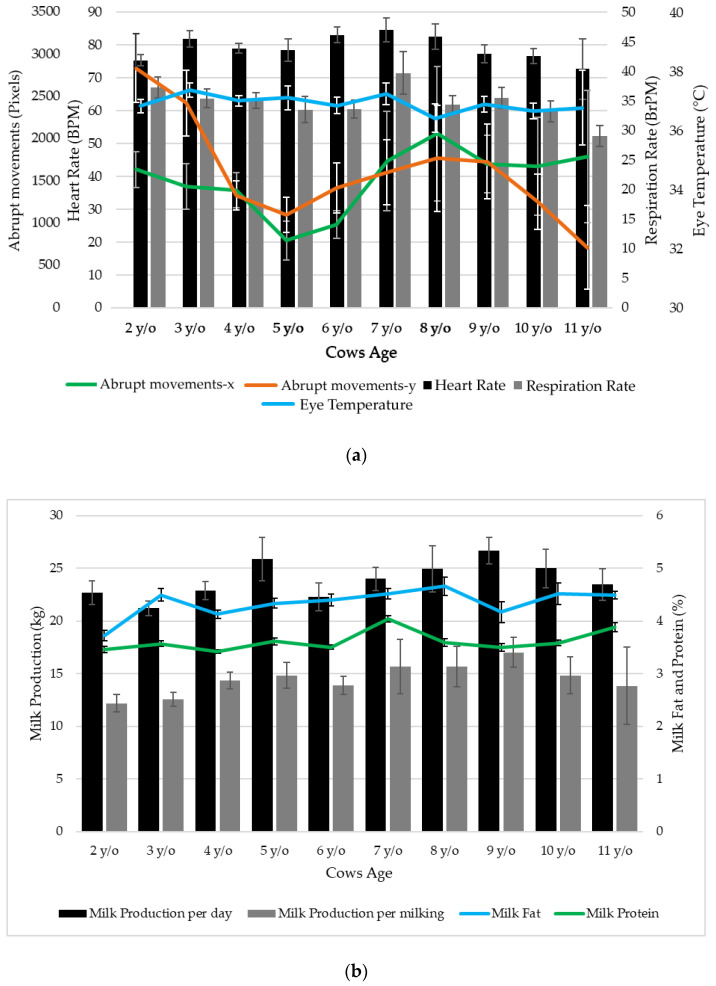
Means and standard error (error bars) of the (**a**) non-destructive physiological responses from cows and (**b**) milk production and composition (fat and protein %) from cows grouped by age. Abbreviations: y/o: years old.

Figure 5a shows that cows within 6 and 8 years old had slightly higher HR (83–85 BPM) than younger or older cows (73–82 BPM), a similar trend was shown for RR of cows 7 years old, which had the highest mean value (40 BrPM) compared to other cows (29–37 BrPM). The abrupt movements in this figure were reported as a variance of x and y axes; therefore, this figure shows that the 5- and 6-year-old cows had fewer movements in both directions (x and y axes) compared to younger and older cows. Furthermore, the most aged cows (11 years old) had low movement in y-, but high on the *x*-axis. On the other hand, all cow age groups had similar mean eye temperature; however, cows 8 years old had slightly lower values.

Figure 5b shows that cows 5 and 9 years old had higher milk production per day (26 and 27 kg, respectively), with cows 3 years old having the lowest (21 kg). While cows within 7 and 9 years old presented the highest milk production per milking (16–17 kg), being the youngest cows (2–3 years old) the lowest in production per milking (12–13 kg). Regarding milk composition, 8-year-old cows produced milk with the highest fat content (4.66%), while 2-year-old cows had the lowest (3.73%). On the other hand, 7-year-old cows had milk with the highest protein content (4.04%) and 4-year-old cows the lowest (3.42%).

Table 1 shows the mean values of the weather data during data collection of cows grouped by age. It can be observed that the mean temperatures at which the cows were tested were within 11 and 13 °C, while the RH was 74–82%. The wind oscillated within 8 and 13 km h^−1^ at 188–243° direction. Comparing the THI calculated with different equations, it can be observed that values were similar, but Equation (S2) (THI1) and Equation (S6) (THI6; Supplementary Material) presented higher values, while calculations with Equation (S10) (THI9) were lower.

**Figure 6:**
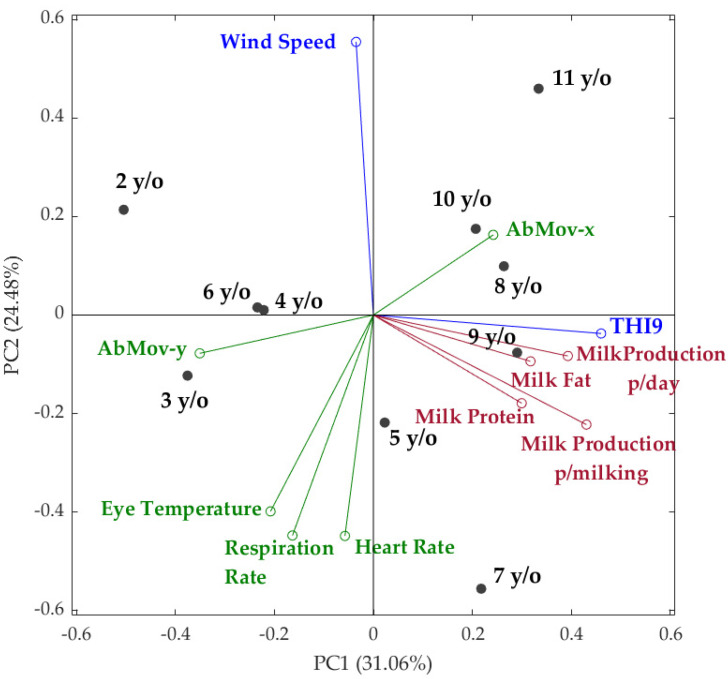
Principal components analysis (PCA) constructed with the physiological (green), weather (blue) and milk (red) parameters. Abbreviations: PC: principal component; THI9: temperature–humidity index calculated with Equation (S10) (Supplementary Material); y/o: years old; AbMov: abrupt movements; p/day and p/milking: per day and per milking.

Figure 6 shows that the PCA accounted for 55.54% of total data variability based on principal components one and two (PC1: 31.06%; PC2: 24.48%). Based on factor loadings (FL), PC1 was mainly represented on the positive side of the axis by THI9 (FL = 0.46) and milk production per milking (FL = 0.43) and by abrupt movements in the *y*-axis (FL = −0.35) on the negative side. On the other hand, PC2 was characterized by wind speed (FL = 0.55) on the positive side of the axis and HR and RR (FL = −0.45) on the negative side. It can be observed that the wind speed was negatively related to eye temperature, RR and HR, while the last three were positively related among them. Furthermore, all milk parameters were positively related to THI9 and negatively related to abrupt movements in the *y*-axis. Older cows (8–11 years old) were grouped and associated with the milk parameters, THI9 and abrupt movements in the *x*-axis, while younger cows 2–4 and 6 years old were grouped on the opposite side from older cows. Middle-aged cows (5 and 7 years old) formed another group and were more associated with the physiological parameters (RR and HR).

### 3.2. Machine Learning Modelling

Table 2 shows that the machine learning model had high overall accuracy (R = 0.96) with high slope values (>0.92) to predict the milk production and quality based on composition (fat and protein) and cows’ eye temperature using the physiological, weather and abrupt movement parameters as inputs. There were no signs of under or overfitting based on the performance values with lower training MSE (9.65) than the testing (MSE = 23.72) stage.

**Figure 7:**
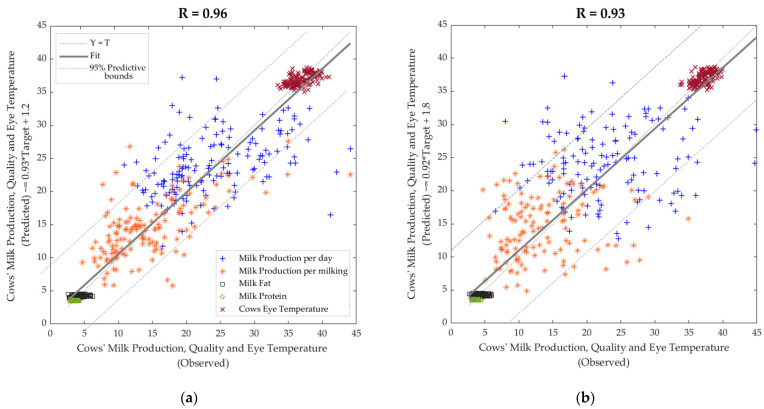
Regression plots from (**a**) machine learning model developed using 150 cow samples and (**b**) deployment using data from 132 cow samples. Abbreviations: R: correlation coefficient.

Figure 7a shows the overall regression model with outliers based on the 95% confidence bounds with 5.5% outliers (41 out of 750 data points). On the other hand, Figure 7b shows the regression model from the deployment using 132 different cow samples. It can be observed that it had high accuracy (R = 0.93) with 8% outliers (53 out of 660 data points).

## 4. Discussion

### 4.1. Cow Physiology, Age and the Environment Related to Milk Productivity and, Quality

The non-invasive HR values reported in this study (Figure 5a) are consistent with averaged HR reported elsewhere using polar sensors during quiet standing of cows (70–90 BPM) [45] and HR variability (5–7 BPM) [46]. In the case of RR, all values recorded were below 60 BrPM, which is related to minimal stress (e.g., thermal stress) [47], which is consistent with THI values for this study (Table 1). Considering averaged THI5 (53, Table 1), the RR presented here (27–43 BrPM) are consistent with those shown elsewhere (20–50 BrPM) for similar environmental conditions [48]. These results are consistent with the machine learning models that estimate human HR and RR [38] and other animals, such as sheep [44]. Eye temperatures for dairy cows are in accordance to those reported as maximum eye temperature in the range of 33–37 °C with the corresponding HR being 60–70 BMP [18] and 36.7–38.3 °C using infrared thermography, which corresponded to around 2 °C less compared to rectal temperatures [49].

Concerning sudden movements, younger cows (2–3 y/o) presented similar and higher sudden movements in x and y based on the error bars compared to older ones (>7 y/o). Previous behavioral research has shown that younger animals head movements may be affected by sudden and novel experiences since they have fewer life experiences than older animals [50].

The milk production trend per day and milking is increasing for 2–5 y/o cows and higher but stabilizing for cows older than 6 y/o; these results are consistent with previous studies [51] (Figure 5b). Milk fat percentages found in this study were also consistent with those reported in the literature, with values of 3.67% for 3 y/o cows compared to 3.80% in this study and 3.48% versus 4.10% for 4 y/o cows, respectively [52]. General protein content in milk has been reported within the ranges of 2.88–4.19% [53], consistent with values reported in this study (Figure 5b).

All the different trends explored before can be seen in the PCA (Figure 6). The THI9 was chosen for multivariate data analysis as it is the most correlated with physiological data and sensitive to describe heat stress in dairy cows [43,54]. In the case of wind, the speed vector is inversely related to physiological parameters, such as HR, RR and eye temperature. Other studies have found that reductions in eye temperature in relation to wind speed are in the order of 0.12 ± 0.02 °C per km h^−1^ (R^2^ = 0.94) [55]. Lower HR and RR related to wind speed are also explained by reduces ambient temperatures [47].

### 4.2. Machine Learning Model Developed and Deployment

Eye temperature was included as a target for machine learning (ML) modeling, along with milk productivity and quality traits (protein and fat content) because it has been frequently reported as the most efficient and accurate parameter acquired using non-contact infrared thermal imagery. The latter required a different camera and computer vision algorithms, which can increase the cost of the technological system proposed. The use of integrated visible and infrared thermal video cameras (IRTV), such as the one used in this study, may help in the development of integrative algorithms [29,56,57] and machine learning modelling [44]. However, FLIR has discontinued the FLIR Duo PRO camera, which complicates the implementation of these models. Inferring eye temperature from RGB video cameras and ML helps avoid incorporating a second camera (IRTV), which can be cost-prohibitive. The cow physiological parameters studied respond to environmental factors and other stressors, such as novel objects/people. It has been shown that eye temperature (related to core body temperature) is regulated mainly through RR and HR [58].

It has been shown that THI is positively related to skin, body surface [59], rectal temperature and RR [60] as a response of cow’s physiology to environmental changing conditions. For milk productivity, cows with higher milk production are positively related to rectal temperature and respiration rate [60]. The latter are consistent with the results presented in this study (Figure 6). Furthermore, HR variability measured using remote sensing techniques accurately measures the autonomic nervous system (ANS) responses to the environment, supporting the idea that HR variability can be used in cattle to measure stress levels and welfare [61]. Hence, RR and HR, which have been remotely measured, are related to the cow’s eye temperature and milk productivity factors, supporting the parameter engineering process used in this research.

Many ML models reported for farm livestock mainly focus on developing and documenting new and emerging technologies and ML algorithms. Furthermore, there has been minimal attention in published work on testing developed ML models and technologies in real-world situations. However, this is consistent with AI implementation in other industries. Only 20% of AI pilots that have been developed for the real world make it to production. However, the latter figures have increased slightly due to COVID-19, and for 2021 are projected to be 20% for machine learning and 25% for AI solutions, according to the Hanover Enterprise Financial Decision Making 2020 report [62]. Incorporating the ML model deployment analysis in this study helps to verify the accuracy of the model developed, considering completely new datasets and measurement days, which rendered similar accuracies (R = 0.93) compared to model development (R = 0.96).

The ML model developed was based only on RGB video data, making it more versatile and applicable to RMS and conventional and small dairy farms. Newly developed AI cameras may facilitate the deployment of these types of models, such as the FLIR (Firefly DL) for deep learning or Firefly S for machine vision with affordable costs. The potential applications of the ML model developed include assessment of animal welfare due to heat stress [43], general health monitoring and disease identification [63,64,65], detection of respiratory diseases [57], biomedical monitoring to optimize cattle treatment [66], drinking behavior [67] and transport conditions [44], among others.

## 5. Conclusions

The industry has a critical requirement to develop and deploy parsimony-based machine learning approaches to assess the welfare and productivity of farm livestock. This research has presented a novel approach to estimate important physiological and productivity information based on visible video cameras that can be affordable, accurate and allow automation. Further studies are scheduled to include data from stressed cows due to heat stress in this model to complement the machine learning model developed. In addition, the models will be tested using different camera brands, specifications, and prices to verify and/or improve accuracy. These models’ applicability is not restricted to robotic dairy farms and can be applied to the conventional dairy farming industry. Furthermore, these methods may benefit not only the dairy farms but also the dairy processing industry as they could guarantee the quality of their milk.

## Figures and Tables

**Table 1:** Mean values ± standard error of weather parameters at the time/date that each cow age group were recorded.

Cows Age	Number of Cows *	Temperature (°C)	Relative Humidity (%)	Wind Speed (km h^−1^)	Wind Direction (°)	THI1	THI2	THI3	THI4	THI5	THI6	THI7	THI8	THI9
2 y/o	42	11.30	81.02	11.84	234.31	62.70	51.13	51.41	56.30	52.51	63.51	55.32	52.51	41.67
		±0.45	±2.33	±0.78	±8.66	±0.58	±0.66	±0.70	±0.58	±0.78	±0.46	±0.46	±0.78	±1.29
3 y/o	46	11.54	79.58	10.89	229.78	63.02	51.50	51.80	56.62	53.02	63.77	55.58	53.01	42.52
		±0.41	±1.96	±0.78	±9.12	±0.53	±0.61	±0.64	±0.53	±0.72	±0.43	±0.43	±0.72	±1.18
4 y/o	63	11.13	81.80	10.51	210.03	62.51	50.94	51.19	56.11	52.37	63.38	55.20	52.37	41.44
		±0.30	±1.49	±0.70	±9.46	±0.39	±0.45	±0.48	±0.39	±0.54	±0.32	±0.32	±0.54	±0.88
5 y/o	19	11.55	80.00	8.34	208.51	63.05	51.55	51.84	56.65	53.03	63.81	55.62	53.03	42.52
		±0.60	±3.29	±1.45	±17.29	±0.77	±0.87	±0.92	±0.77	±1.05	±0.60	±0.61	±1.05	±1.75
6 y/o	38	11.01	81.47	11.26	227.73	62.32	50.69	50.96	55.92	52.13	63.20	55.01	52.13	41.09
		±0.46	±2.01	±0.91	±10.18	±0.60	±0.71	±0.73	±0.60	±0.82	±0.50	±0.50	±0.82	±1.34
7 y/o	10	12.58	75.19	8.56	188.28	64.39	53.09	53.46	57.99	54.92	64.89	56.71	54.91	45.61
		±0.62	±3.63	±1.63	±31.37	±0.78	±0.88	±0.93	±0.78	±1.07	±0.60	±0.61	±1.07	±1.79
8 y/o	14	12.01	77.26	11.66	223.71	63.62	52.19	52.53	57.22	53.86	64.25	56.07	53.85	43.91
		±0.74	±3.23	±1.48	±13.81	±0.97	±1.12	±1.17	±0.97	±1.28	±0.78	±0.79	±1.28	±2.08
9 y/o	24	12.88	74.51	10.13	221.26	64.79	53.58	53.96	58.39	55.42	65.25	57.07	55.41	46.39
		±0.35	±2.08	±1.06	±10.05	±0.44	±0.49	±0.52	±0.44	±0.59	±0.33	±0.34	±0.59	±1.00
10 y/o	23	12.04	77.82	11.28	232.96	63.70	52.32	52.64	57.30	53.99	64.35	56.17	53.99	44.09
		±0.46	±2.26	±0.89	±11.32	±0.59	±0.68	±0.71	±0.59	±0.81	±0.48	±0.48	±0.81	±1.33
11 y/o	3	12.57	75.10	13.06	243.30	64.39	53.11	53.47	57.99	55.04	64.90	56.73	55.03	45.83
		±0.52	±4.30	±3.50	±7.56	±0.61	±0.64	±0.71	±0.61	±0.88	±0.40	±0.40	±0.88	±1.55

Abbreviations: y/o: years old; THI: temperature–humidity index. The number next to the THI corresponds to the calculations from different equations in the Supplementary Material. * Including replicates. Multivariate Data Analysis.

**Table 2:** Statistical data from the machine learning model showing the correlation coefficients (R) and performance based on means squared error (MSE).

Stage	Samples	Observations	R	Slope (b)	Performance (MSE)
Training	105	525	0.97	0.94	9.65
Testing	45	225	0.93	0.92	23.72
Overall	150	750	0.96	0.93	-

## Data Availability

Data and intellectual property belong to The University of Melbourne; any sharing needs to be evaluated and approved by the University.

## Supplementary Materials

The following are available online at https://www.mdpi.com/article/10.3390/s21206844/s1. Table S1. Summary of methods to measure physiological responses of animals including advantages and disadvantages; Equations (S1)–(S10); Figure S1. Diagram depicting the process to be followed for deployment of the proposed integrated system. Boxes in the cow represent the area of interest for each assessment, x and y represent the abrupt movement assessment in x and y axes.
