# Supplementary material for: Biometric Physiological Responses from Dairy Cows Measured by Visible Remote Sensing Are Good Predictors of Milk Productivity and Quality through Artificial Intelligence
Source: Sensors (Basel). 2021 Oct 14;21(20):6844. doi: 10.3390/s21206844 (PMC8541531; doi:10.3390/s21206844)
Supplement: Supplementary file 1 [file sensors-21-06844-s001.zip › sensors-1384359-supplementary.pdf]

Table S1. Summary of methods to measure physiological responses of animals including advantages and disadvantages

Measurement	Technique	Advantages	Disadvantages	References
**Heart Rate (HR)**	Wearable sensors	Traditional methods used as groundtruth. Effective for single/few measurements.	Increase awareness and stress, which may alter the readings	[23-26]
	Contactless biometrics Computer vision	Non-invasive, reduces stress, continuous monitoring, no human intervention	Requires recording ~10 s of animal's face/eyes	[27,46]
**Respiration Rate (RR)**	Manual count (visual + chronometer)	Traditional methods used as groundtruth Effective for single/few measurements	May lead to human error Time consuming	[27,28]
	Contactless biometrics Computer vision	Non-invasive, reduces stress, continuous monitoring, no human intervention	Requires recording ~10 s of animal's face/nose	[27,46]
**Body Temperature**	Thermometers / Wearable sensors	Traditional methods used as groundtruth Effective for single/few measurements	Increase awareness and stress, which may alter the readings	[29-32]
	Infrared thermal images	Non-invasive, reduces stress, continuous monitoring, no human intervention May assess temperature in different parts of the body	Requires high investment in thermal cameras	[18,27,42,46]
	Computer vision and machine learning using RGB videos	Low investment as may use any RGB camera Non-invasive, reduces stress, continuous monitoring, no human intervention	Requires recording of either eyes or any similar hotspot in cows body	More details in Materials and Methods and Results
**Proposed system**	Computer vision and machine learning proposed in this paper	Measure physiological responses (HR; RR; abrupt movements) and Prediction of eye temperature, milk production and composition. May feed new data to improve accuracy. It does not need of extra infrared cameras including less computation	Requires animals passing through a single determined path per camera system. Sites without internet access need *in situ* processing components (e.g. Jetson NVIDIA Nano).	More details in Discussions

$$T_{dp} = \frac{243.5\left(\frac{17.67\times T}{243.5+T}+ln\frac{RH}{100}\right)}{17.67-\left(\frac{17.67\times T}{243.5+T}+ln\frac{RH}{100}\right)} \tag{S1}$$

$$THI_1 = 0.4 \times (T + T_{wet}) \times 1.8 + 32 + 15 \tag{S2}$$

$$THI_2 = (0.15 \times T + 0.85 \times T_{wet}) \times 1.8 + 32 \tag{S3}$$

$$THI_3 = (T \times 0.35 + T_{wet} \times 0.65) \times 1.8 + 32 \tag{S4}$$

$$THI_4 = 0.72 \times (T + T_{wet}) + 40.6 \tag{S5}$$

$$THI_5 = (1.8 \times T + 32) - [(0.55 - 0.0055 \times RH) \times (1.8 + T - 26)] \tag{S6}$$

$$THI_6 = \left(0.55 \times T + 0.2 \times T_{dp}\right) \times 1.8 + 32 + 17.5 \tag{S7}$$

$$THI_7 = T + \left(0.36 \times T_{dp}\right) + 41.2 \tag{S8}$$

$$THI_8 = (0.8 \times T) + \left(\frac{RH}{100}\right) \times (T - 14.4) + 46.4 \tag{S9}$$

$$THI_9 = 3.43 + 1.058 \times T - 0.293 \times RH + 0.0164 \times T \times RH + 35.7 \tag{S10}$$

Abbreviations:

T_{dp}: Dew point temperature

T: Temperature

RH: Relative humidity

THI: Temperature humidity index

T_{wet}: Wet temperature

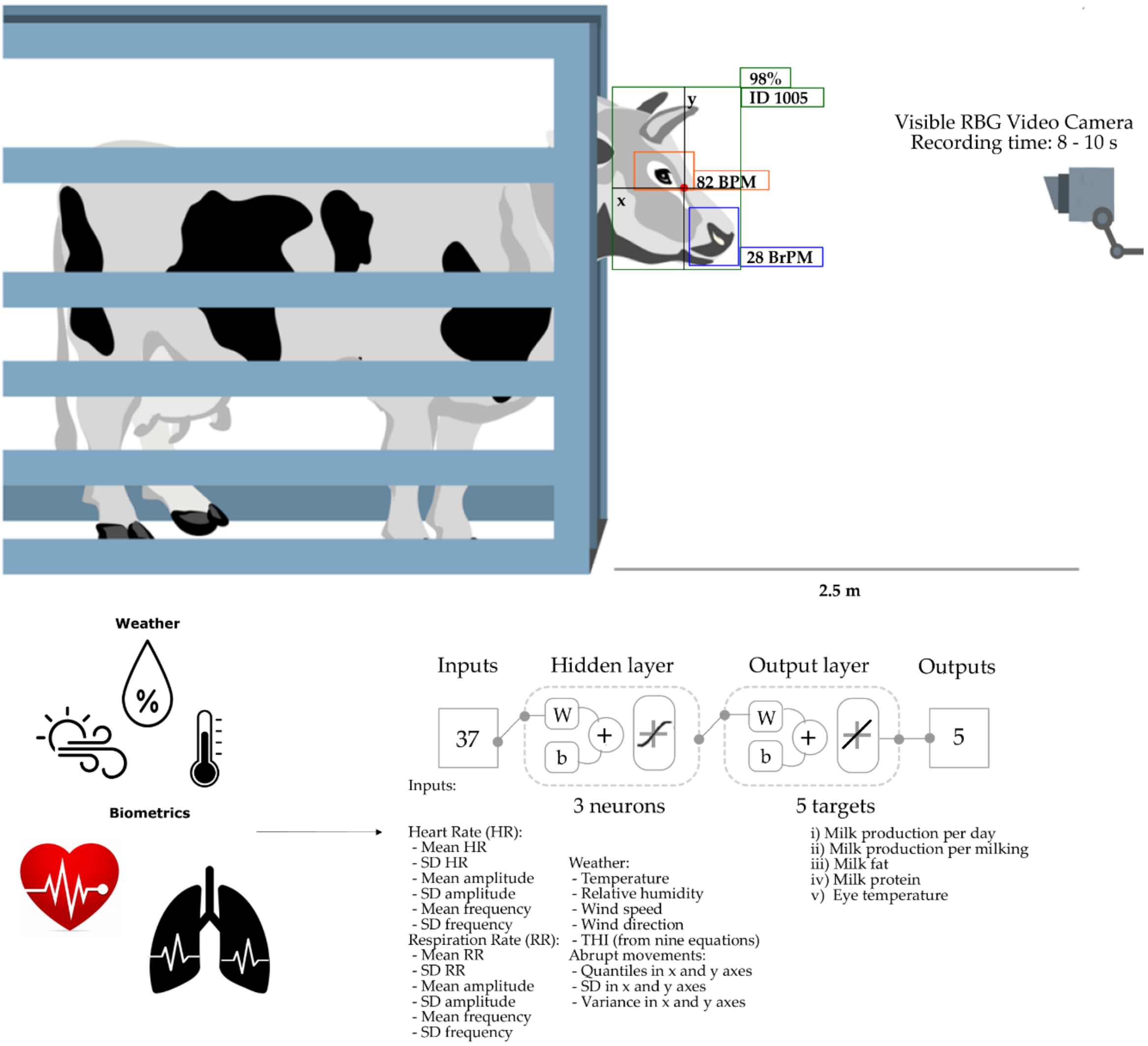

Figure S1. Diagram depicting the process to be followed for deployment of the proposed integrated system. Boxes in the cow represent the area of interest for each assessment, x and y represent the abrupt movement assessment in x and y axes.
